# MicroRNAs Role in Breast Cancer: Theranostic Application in Saudi Arabia

**DOI:** 10.3389/fonc.2021.717759

**Published:** 2021-10-25

**Authors:** Nouf M. Alyami

**Affiliations:** Department of Zoology, College of Science, King Saud University, Riyadh, Saudi Arabia

**Keywords:** chemotherapy resistance, breast cancer metastasis, molecular pathways, anticancer therapy, Saudi Arabia, miRNA, circulating biomarkers

## Abstract

Breast cancer is an aggressive silent disease, representing 11.7% of the diagnosed cancer worldwide, and it is also a leading cause of death in Saudi Arabia. Consequently, microRNAs have emerged recently as potential biomarkers to diagnose and monitor such cases at the molecular level, which tends to be problematic during diagnosis. MicroRNAs are highly conserved non- coding oligonucleotide RNA. Over the last two decades, studies have determined the functional significance of these small RNAs and their impact on cellular development and the interaction between microRNAs and messenger RNAs, which affect numerous molecular pathways and physiological functions. Moreover, many disorders, including breast cancer, are associated with the dysregulation of microRNA. Sparingly, many microRNAs can suppress cancer cell proliferation, apoptosis, angiogenesis, invasion, metastasis, and vice versa. Remarkably, microRNAs can be harvested from patients’ biofluids to predict disease progression that considered a non-invasive method. Nevertheless, MicroRNAs are currently utilized as anti- cancer therapies combined with other drug therapies or even as a single agents’ treatment. Therefore, this review will focus on microRNAs’ role in breast cancer as an indicator of disease progression. In addition, this review summarizes the current knowledge of drug sensitivity and methods in detecting microRNA and their application to improve patient care and identifies the current gaps in this field.

## 1 Introduction

Breast cancer (BC) is the dominant type of cancer among female patients, reaching 2,261,419 new cases in 2020, representing 11.7% of the yearly diagnosed patients with cancer worldwide. BC incidence has declined dramatically in industrial countries, except for Australia/New Zealand and Western Europe (1). Despite the advances in BC diagnosis, the leading cause of mortality is the disease recurrences due to metastases. Management of disease recurrences and metastasis has modestly improved over the last three last decades (2). Metastasis states the spread of cancer cells through the lymphatic system or bloodstream to distant organs (3). Because of these challenges, the need for sufficient molecular biomarkers to predict the disease response is continued. However, researchers are examining the utility of MicroRNAs as biomarkers to detect diseases and tumor aggressiveness (4, 5).

MicroRNAs (miRNAs) were discovered in the 1990s in nematodes (6, 7). miRNAs are approximately 19–25 nucleotides (nt) in length and are found in almost all eukaryotes. Since then, many studies have identified miRNAs’ functionality and role in disorders and human illnesses such as BC (4). miRNAs can regulate genes by silencing their protein-coding mRNA (messenger RNA) through inducing mRNA turnover. miRNAs are determined to be involved in cellular activities such as tumorigenesis, proliferation, cell survival, apoptosis, and cancer development, affecting cancer progression (5). These small oligonucleotides can function as oncogenes by degrading mRNAs that act as tumor suppressors and *vice versa.* Previous studies showed that many miRNAs impacted Breast cancer development and even drug resistance (8, 9). Due to the heterogeneous nature of the BC, it is considered a challenge, which makes it extremely difficult to classify and treat (2). Concomitantly, many countries, specifically Saudi Arabia, are suffering from recurrent disease conditions due to metastases.

Nonetheless, using blood serum and non-invasive methods that are considered safe and accurate to determine the molecular characterization and create a personalized treatment strategy for each patient to prevent recurrence in the future had been utilized. Therefore, this review focuses on miRNAs’ role in breast cancer, wherein they serve as biomarkers to detect tumors, including their progression, treatment resistance, and potential impact on clinical practices.

## 2 Manuscript Formatting

### 2.1 Background

Ambros and Ruvkun laboratory discovered the first miRNA and its target in 1993. Ambros’s lab has found the *lin-4* gene a fundamental player in *Caenorhabditis Elegans* (*C. elegans*) development. However, the *lin-4* gene does not encode any known cellular protein, but it only generates a short 22 nt RNA. Furthermore, the Ruvkun lab has determined that this small RNA sequence is complementary to the 3′UTR (3’untranslated region) and negatively regulates the *lin-14* gene (6, 7). Seven years later, let-7, a small 21-nt RNA, was discovered and was further identified in various species (10, 11). Since then, thousands of miRNAs and their genetic functions in humans and other animals have been identified (4, 5).

Interestingly, large projects such as FANTOM and ENCODE for genomic annotation and functionality have reported that 80% of mammalian DNA is actively transcribed. The vast majority are noncoding RNA genes (ncRNA) (12, 13). In the past, the main differences between coding and non-coding were based on encoding protein. However, this barrier starts to overlap as particular coding RNA, such as TP53 mRNA, can function as RNA only, significantly impacting much biologic development (14, 15). Furthermore, long non-coding RNA (lncRNA) can regulate gene expression at both genomic and post-transcription levels. At the genomic level by manipulating chromatin status and complementary binding to other forms of RNA such as miRNA and mRNA as a post-transcription level (16–19). Also, other studies identified that lncRNA could encode small peptides, but their functions are still unknown (20, 21). Other types of RNA that also function similarly to miRNAs with the exact mechanism (using cytoplasmic processing proteins) are the small interferences RNA (siRNA) (22). They can silence gene expression as miRNA *via* targeting the mRNA but not expressed endogenously as miRNA encoded in the genome. Plus, they can only target one specific mRNA, as for miRNA that can have vast mRNA targets (9, 23). The source of these siRNAs can be viruses as they can manipulate the host gene expression using this tool (23). Nearly 3% of the human genome encodes miRNA genes. These small RNAs play a critical role in various biological processes such as cell apoptosis and development in plants and animals. They function at the translational or mRNA degradation stages (24). Additionally, more than 60% of the *Homo sapiens* mRNA-coding proteins with putative binding sites for miRNA were predicted (25). More than 2,654 mature miRNAs and 1,917 precursor miRNAs are listed for *Homo sapiens*, as reported on the miRBase database (26).

### 2.2 MicroRNA Biogenesis and Biology

RNA polymerase II (Pol II) generates a transcript identified as pri-miRNA (primary miRNA) during the transcription of the genomic miRNAs in the cell nucleus. Spliced introns of protein- coding genes give rise to approximately 30% of miRNAs. However, most miRNAs encoded gene loci or clusters in the genome. First, the pri-miRNAs comprise more than 1000 bases and stem- loop/hairpin structures with a cap and poly-adenylated UTRs. Second, these UTR modifications are cleaved into pre-miRNAs (precursor miRNAs) with 60 to 110 nt by Drosha and DGCR8/Pasha proteins. Pre-miRNAs reportedly binds to XPO5 (Exportin-5) to translocate to the cytosol. The pre-miRNAs are then cleaved by Dicer, generating 15 to 22 nt short double-stranded miRNA duplexes. Finally, DICER and Argonaute (AGO) proteins disassemble the miRNA duplex because of their endoribonuclease activity.

**Figure 1:**
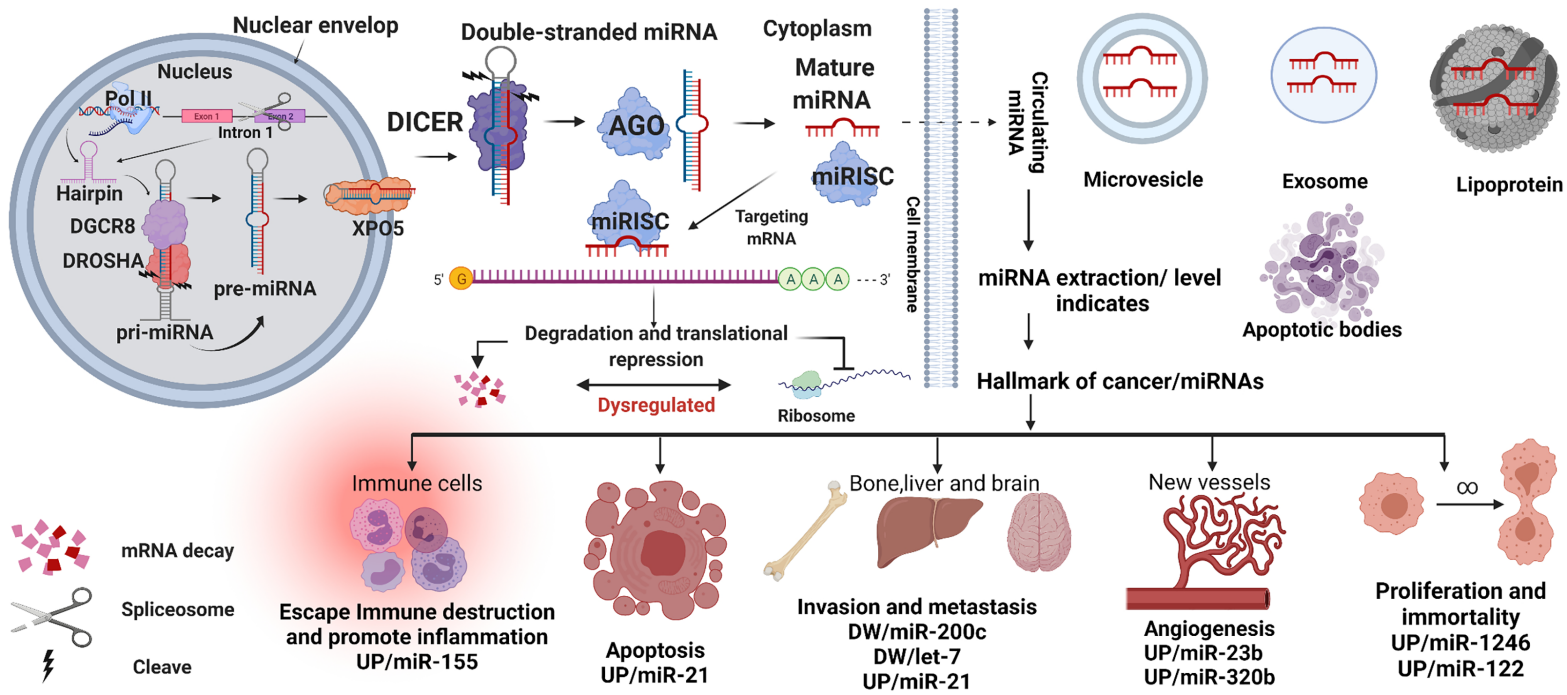
MiRNA biogenesis. miRNA genes or spliced introns are transcribed by polymerase II (Pol- II) as primary miRNA (pri-miRNA). The pri-miRNA is subsequently cleaved by Drosha along with DGCR8 proteins to generate the pre-miRNAs. The pre-miRNAs are then exported to the cytoplasm by Exportin-5 (XPO5) and cleaved again by DICER1 to form a short double- stranded miRNA. Together with Argonaute (AGO), this double-stranded miRNA is unwound into a mature miRNA (single strand) and loaded as a guide for the miRNA-induced silencing complex (miRISC) to target the UTRs or CDSs of the mRNA. Based on the mature RNA sequences, miRISC could repress mRNA expression. The mature miRNA can export from the cell and reaches the bloodstream as lipoproteins, exomes, microvesicles, and apoptotic bodies. Which can be used as a marker for cancer-based on their expression levels, UP or DW (down)—created with BioRender.com.

Interestingly, viruses can hijack this process and eventually manipulate the host’s gene expression by mimicking the host’s short double-stranded miRNA (23). Subsequently, a single strand, called mature miRNA, is assembled into the miRNA-associated RNA- induced silencing complex (miRISC), including DICER and AGO. The miRISC complex can target the UTRs or the coding sequences (CDSs) based on the RNA strand sequence, as illustrated in **Figure 1**. In addition, the miRISC complex suppresses the protein synthesis genes or degrades the mRNAs. The complete alignment with the target mRNA leads to its degradation, whereas the incomplete alignment leads to translation suppression, as shown in **Figure 1** (27).

Furthermore, the mature miRNAs can also exit the cell by different packaging systems. For example, the identification of exosomes containing miR-23b, miR-320, miR-21, let-7a, and miR- 1246 are elevated in plasma patients with breast cancer and used as markers for cancer (28, 29). In addition, other proses like lipoproteins, microvesicles, and apoptotic bodies as displayed in **Figure 1** (30–32).

### 2.3 Methods on Isolating MicroRNAs

Over the last decade, thousands of studies covering miRNA-related discoveries and published. Recent studies have investigated miRNA’s role in autoimmune, cardiovascular, and neurological diseases and cancer. Additionally, these studies have identified novel approaches for collecting miRNA from serum to detect metastases and disease prognosis in cancer patients (4). The main goal is to use these miRNAs as biomarkers to develop a fast, non-invasive clinical test for disease diagnosis and prognosis. Many well-known companies currently provide isolation kits using the body biofluids to collect these small circulating non-coding RNA and methods for quantifying them. One study compared six commercial kits and used fresh, frozen, and low volumes of serum to detect sensitivity (33). Another study used serum and cerebrospinal fluids to compare the commercial kit and TRlzol extraction methods for miRNAs recovery and found that TRlzol isolation techniques have low recovery (34). However, many other studies added suggestions and modifications to the TRlzol extraction methods and improved the recovery of the circulating miRNAs over commercial kits (35–37). Quantification of the isolated biomarkers can be performed using *NanoDrop spectrophotometers.* Then using quantification polymerase chain reaction (qPCR) after generating the complementary DNA (cDNA) and for novel miRNA usually using microarray and deep sequencing (34).

### 2.4 Bioinformatics Analysis

Microarray, qPCR, Next generation-sequencing advancing technologies are becoming more feasible, making them less expensive than before to quantify miRNA; however, it is only the beginning of any project. Thus, the main challenges are identifying miRNA candidates and their functionality, coding-protein gene targets, and molecular network pathway. Bioinformatics analysis predicts the possible targets for miRNA based on the giving sequences using a specific algorithm. Many databases are available online and always competing on their updates and algorithms. For instance, TargetScan uses the miRNA seeds, which are unique sequences for miRNA, to calculate all the possible binding sites and strength to inhibits the mRNA. Other databases predict targets, such as TarBase, PicTar, and miRBase. Identifying the biological activity and pathways is also available, like; GO (Gene ontology), KEGG, and STRING (38).

### 2.5 Cancer and MicroRNA

The first study to demonstrate a connotation between miRNAs and tumors was published in 2002. The authors reported that miR-16-1 and miR-15 gene deletions were common in chronic lymphocytic leukemia (CLL) (39). Their expression is inversely correlated with the anti-apoptotic protein and B-cell lymphoma protein (Bcl-2) expression. Both miR-16-1 and miR-15 expressions act as tumor suppressors by suppressing Bcl-2 expression, leading to the induction of apoptosis of leukemic stem cells (40, 41). Interestingly, Bcl-2 was recognized as a suitable biomarker for the prognosis of all molecular subtypes of BCs (42), indicating the potential role of miRNAs in BCs diagnosis. Hence, somatic inhibition of miR-16-1 and miR-15 stimulates leukemogenesis and inhibits cell death (40, 41). Abnormal and dysregulation of miRNA functions have been described in several other cancers, such as lung, breast, colorectal, and leukemia. miRNAs are classified into tumor suppressors or oncogenes (also named oncomir). For example, the miR-30 family, miR-16- 1, miR-15, and miR-34 considered tumor suppressors, whereas miR-10, miR- 155, and miR- 200 family act oncogenes (43). The deregulation of oncomirs or tumor suppressor miRNAs can induce tumorigenesis by manipulating molecular pathways to promote cancer hallmarks, such as proliferation, inhibition of apoptosis, invasion, resistance, and angiogenesis, leading to tumor survival and metastasis (44). Although miRNAs can act as oncomirs or tumor suppressors, studies have also suggested that the global loss of miRNAs can augment tumor progression. Therefore, miRNA dysregulation can promote cellular transformation and carcinogenesis with Dicer, Drosha, and DGCR8 mutation (45).

### 2.6 MicroRNAs as Diagnostic Marks in Breast Cancer

BC has possible risk factors and lifestyle, family history (genetic alteration in the *BRCA1* and *BRCA2*), age, weight, exposure to radiation, and hormones. In addition, there are two common breast carcinoma types; these are ductal and lobular. Consequently, the treatment strategies are adjusted based on the disease type. Currently, BC is genetically subclassified based on estrogen hormone receptors’ levels, human epidermal growth factor receptor (HER2), which can determine the treatment choice (46).

Because BC is remarkably heterogeneous and classified into several subtypes, treatment response and prognosis prediction are challenging. Therefore, new biomarkers are needed (2). Dysregulation of miRNA was associated with many disorders, including BC. Ongoing studies examine miRNA profiling as a strategy to predict disease progression, improve patient survival, and develop new BC classification strategies (47). Using miRNA expression as a fingerprint would enhance our understanding of disease heterogeneity and novel therapeutics’ molecular development. For instance, the expression levels for miRNA cluster miR- 125b/miR-99a/let-7c were used as markers to identify luminal A and B subtypes; further, it was correlated with luminal A patients’ survival rates (48). Additionally, HER2-encoded miR-4728 expression was precise to detect tumors that are enriched with HER2 receptors. Another cluster, miR-96/182/183, was reported by Zhang et al. and was found to enhance epithelial-to-mesenchymal transition (EMT), which can cause BC cells to be more invasive (49).

Since the 2000s, many more miRNAs have been discovered and linked with BC’s development and initiation (50), as described in **Table 1**. Some of the most recognized miRNA families are let-7, miR-200, and miR-10.

**Table 1:** Summary of miRNAs associated with drug sensitivity and prognosis in breast cancer.

miRNA	Prognosis	Pathways/Genes	Drug sensitivity/resistance
miR-187-5p and miR-106a- 3p	H,PR (51, 52)	HIPK3 and EGFR pathway	Resistant to taxanes, paclitaxel, and docetaxel (53, 54)
miR-182-5p	H,PR (55)	Cx43	Resistant to veliparibv (53, 54)
miR-629-5p	H,PR in NSCLC (56)	FOXO3, CXXC4, SFTPC	Resistant to tipifarnib (53, 54)
miR-637	H,PR (57)	Akt1/*ββ*-catenin (cyclin D1) pathway	Resistant to tivantinib (53, 54)
miR-556-5p	H,GR (58)	YAP1	Sensitive to paclitaxel (53, 54)
let-7d-5p and hsa-miR-18a-5p	H,PR (59, 60)	Wnt pathway and BSG	Sensitive to tivantinib (53, 54)
let-7a-5p	H,PR (61)	MYC,HMGA2, H-RAS, HMGA2, DUSP7	Sensitive to bortezomib and paclitaxel (61)
miR-135a-3p	H,PR (62)	HOXA10	Sensitive to JNJ-707 (53, 54)
miR-185-3p	H,GR (63)	E2F1	Sensitive to panobinostat (53, 54)
miR-449	H,GR (64)	TPD52	Sensitive to Doxorubicin (65)
miR-140	H,GR (66)	Wnt1 pathway	Sensitive to fluorouracil, cisplatin, doxorubicin, paclitaxel, and camptothecin (66, 67)
miR-130b	H, PR	PI3K/Akt pathway	Resistant to adriamycin, vincristine, and paclitaxel (68)
miR-29a	H, PR (69)	TET1 and PTEN/AKT/GSK3β pathway	Resistant to adriamycin (70)
miRNA-132 and miRNA- 212	H, PR	PTEN/AKT/NF-KB pathway	Resistant to doxorubicin (71)

High expression (H), Poor Respond (PR), Good Respond (GR), Homeodomain Interacting Protein Kinase 3 (HIPK3), Epidermal Growth Factor Receptor (EGFR), Connexin 43 (Cx43), Non-small-cell lung cancer (NSCLC), Forkhead Box O3 (FOXO3), CXXC Finger Protein 4 (CXXC4), Surfactant Protein C (SFTPC), Yes1 Associated Transcriptional Regulator (YAP1), Basigin (BSG), Homeobox A10 (HOXA10), Tumor Protein D52 (TPD52), Ten Eleven Translocation 1 (TET1).

The family of let-7 miRNAs in humans includes ten members known to function as tumor suppressors, and they have miR-202, miR-98, and let-7a, b, c, d, e, f, g, and i (72). Let-7 targets multiple molecular pathways contributing to BC heterogeneity and metastases by activating the cancer stem cell (CSC) phenotype (73). On the one hand, a clinical study found the expression of let-7 was considerably lower in patient’s serum with BC that developed metastases (74). On the other hand, using Saudi plasma, let-7b-5p, hsa-let-7c-5p, and hsa-let-7i-5p miRNAs were elevated in luminal BC patients and triple-negative BC samples except hsa-let-7c-5p compared to the control (75).

The self-renewing, undifferentiation, and chemotherapy resistance abilities are key CSC features found in BC tumor-initiating cell lines (T-IC). Furthermore, let-7 targets the 3’UTR of HMGA2, a high-mobility group protein, and H-RAS mRNA. T-IC cell lines have shown significant expression of both targets due to the loss of let-7 activity. These targets’ expression was reduced upon transfection of T-IC cell lines with let-7 lentiviruses (73, 76). Other well-known oncogenes are also targeted by the let-7 family, such as MYC (Myelocytomatosis), KRAS, NRAS, CDK6 (Cell division protein kinase 6), and CdC25 (Cell division Cycle) (77–79).

The second family is miR-200, consisting of miR-200a, b, c, miR-429, and miR-141. These miRNAs regulate the cell self-renewal *via* B lymphoma Mo-MLV insertion region 1 homolog (BMi1), a known oncogene. This protein, BMi1at high levels, inductees the cell transformation of mammary cells to BC stem cells (80). Furthermore, a report by Jurmeister demonstrated that miR-200c modulates cellular movements. The expression level of miR-200c has been determined to correlate negatively with formin homology 2 domain containing 1 (*FHOD1*) and protein phosphatase, Mg^2+^/Mn^2+^-dependent, 1F (*PPM1F*) levels which are known to promote EMT in BC cell lines by modulating actin formation (81). The ability to move is a sign of aggressiveness, explaining the loss of miR-200c serum in patients diagnosed with triple-negative BC (82).

The third family is miR-10, which was dysregulated in several human cancers, including BC (83). In BC patients, miR-10a was significantly overexpressed in primary tumor samples and cell lines (84). Additionally, the high expression of miR-10b is associated with highly metastatic BC cell lines and in patients with lymph node metastatic (85). In contrast, a study by Ma et al. reported no significant correlation between miR-10b levels and BC patients with distant metastasis (86, 87).

Moreover, many individual miRNAs were also found to interact directly or indirectly with key molecular pathways such as oncomirs or tumor suppressors, modulating BC tumorigenesis. One of the most exceedingly expressed miRNAs in BC has been identified as the oncomir miR-21, which plays a critical role in cancer apoptosis, initiation, migration, and invasion; furthermore, it correlates with tumor development and poor outcomes (88, 89). Such as the significant diagnostic power for miR-21 for BC prediction using Egyptian serum (90). Remarkably, miR-21 targets and suppresses signal transducers and activators of transcription 3 (STAT3) mRNA. Interestingly, STAT3 elevation is an essential biomarker for early detection of 220 BC (8, 91).

MiR-155 is another oncomir that controls many pathways associated with proliferation and reduced survival rates by targeting *BRCA1*, which was identified to play a part in DNA repair and initiation of BC and cell cycle progression (92). Furthermore, miR-155 expression correlates with BC metastasis (93). MiR-155 was also reported to affect apoptosis pathways through caspase 3 by repressing the tumor suppressor gene suppressor of cytokine signaling (SOCS1). Additionally, the activation of miR-155 in BC results in the constitutive stimulation of STAT3 through the JAK network. This pathway induces interleukins and interferons’ production, leading to an inflammatory response in BC development (94). This correlated with the circulating miRNA in mice plasma with breast cancer that decreased significantly when introducing an anti-drug agent miR-155 that reduced inflammation and tumor growth (95). In 2020, a study collected the circulating miR-155 from BC patients and controls that predicted the disease even the grade type (96).

Another miRNA that is often silenced in BC is miR-335, which suppresses all cancer phenotypes except proliferation. miR-335 inhibits metastasis by inhibiting the extracellular matrix protein tenascin-C and transcription factor SOX-4 (97). In addition, miR-335 can reduce cell viability and enhance cell death by modulating the BRCA1 activator network as a metastasis suppresser. However, BRCA1 mutation is the primary pathogenesis for BC and is already nonfunctional even when upregulating miR-335 (98).

Meanwhile, miR-34a is one of the most studied miRNAs that acts as a tumor suppressor and a miR-34b and miR-34c family (99–102). Through targeting silent information regulator 1 (SIRT1), miR-34a induces cell cycle arrest, apoptosis, inhibition of EMT, and proliferation of CSCs (99). Besides, miR-34a targets multiple genes, including Fra-1, LMTK3, Bcl-2, and Notch, implicated in BC tumorigenesis. Although accumulating evidence indicates that miR-34a acts as a tumor suppressor, the suppression of miR-34a was found to promote docetaxel resistance in MCF-7 cells, a known docetaxel-resistant cell (100). However, miR-34a is frequently repressed in BC, which supports BC proliferation and survival (101). Furthermore, this family can also target the mRNA of SIRT1 (silent mating type information regulation 2 homolog) and MYC (102).

MiR-205 is also repressed in metastatic BC Deregulation of miR-205 enhances BC cell invasion and proliferation (103). The expression of miR-205 was found to inhibit cell growth, clonogenic survival, and enhancement of response to tyrosine kinase suppressors and anchorage- independent cell growth with HER3 (104).

### 2.7 MicroRNAs as Prognostic Marks in Breast Cancer

Predictive factors give information on whether a patient with cancer will respond to treatment; these are also further used to predict the risk of developing diseases. Unfortunately, despite the marked advances in cancer treatment, chemotherapy resistance remains a significant challenge. Thus, a better comprehension of drug resistance mechanisms is necessary to enhance treatment outcomes. Many factors are associated with drug resistance, such as multidrug resistance protein 1 (MDR1), DNA repair pathways, cell death, and epigenetic modification (105). miRNA can interfere with drug targets that regulate cell survival, apoptotic signaling, and DNA repair pathways. Moreover, miRNAs could modulate cellular responses to anti-cancer treatments (106). Nowadays, prognostic or predictive factors have tremendous potential as biomarkers to guide cancer treatment options. Prognosis predicts the development and disease outcomes and their impact on life quality (107). The most common dysregulated circulating miRNAs are also found in body fluids such as blood. For example, hsa-mir-3662, hsa-mir-19a, hsa-mir-210, and hsa- mir-7 are located in seven types of cancers. These miRNAs have been determined to significantly impact cancer progression because they regulate critical pathways such as mitogen-activated protein kinases, apoptosis, phosphatidylinositol 3-kinase (PI3K), and Akt/protein kinase B (108).

Interestingly, global dysregulation of miRNAs in many types of cancer can serve as a key prognostic factor. For instance, Dicer and Drosha expression loss are critical in miRNA biogenesis and correlated with poor survival in cancer patients (45).

Collectively, this growing evidence indicates that miRNA profiling and miRNA involvement in drug resistance could help choose the right treatment strategies that most likely will lead to positive outcomes for cancer patients (106). Such as identifying eight miRNAs that can be used as a prognosis after surgery and treatment for triple-negative BC to predict recurrently. They are, miR-20a-5p, miR-455-3p, miR-486-5p, miR-146b-5p, miR-107, miR-324-5p, miR-139-5p and miR-10b-5p (109).

Furthermore, Li et al. identified miR-210 as a therapeutic utility as a biomarker for BC recurrences (110). In 2020, miR-622, a novel miRNA, coupled with poor survival in patients with BC (111). Interestingly, miR-622 was isolated from the patients’ plasma in these studies, representing a fast and non-invasive diagnostic method. Similarly, miR-4317 was correlated with lymph node metastasis when it is down-regulated. Sheng et al. used meta-analysis and found candidate targets for miR-4317, and MYD88 mRNA was negatively correlated with a miR-4317 inhibitor that demolished the BC cell lines’ ability to migrate, invite, and proliferate shown a significant biomarker value for prognosis (112). Finally, a study demonstrated the potential use of miRNA as an indicator for drug sensitivity and investigated 114 miRNAs and chemotherapy sensitivity in 36 BC lines, as displayed in **Table 1** (53). Also, we integrated the prognosis factor for each of these miRNA using BC patient samples.

### 2.8 MicroRNAs Reported in BC Patients From Saudi Arabia

BC is still considered a significant disease that affects women, even in developed countries, including Saudi Arabia. More than 1.9 million women are estimated to have BC in 2020, which increased by 18.4% from 2012 (113). According to the Global Cancer Observatory 2018, BC ranked as the most common cancer in Saudi Arabia in both genders; however, it is more common among females. Additionally, BC was identified as the second leading cause of death after leukemia (113). The incidence rate of BC reported between 2010 and 2017 among females ranged from 3 to 8 confirmed cases out of 1000 admitted patients to the Armed Forces Hospital Southern Region, recording the highest rate in 2017 (114). The major cause of death in Saudi BC patients is distal metastases, representing 44.92%, followed by regional metastasis 42.92%; it was determined that 12.15% of deaths had localized diseases (115). These results further highpoint the need for improved screening methods. Qattan et al. used a non-invasive method to isolate circulating miRNAs from Saudi female BC patients’ plasma. They identified five significantly elevated miRNAs compared to the control groups. These miRNAs included hsa-let-7i-5p, hsa- miR-25-3p, hsa-miR-16-5p, hsa-let-7b-5p, and hsa-miR-199a-3p. Furthermore, hsa-let-7b-5p, hsa-let-7c-5p, and hsa-let-7i-5p miRNAs were determined to be specifically elevated in luminal BC patients and triple-negative BC samples except for hsa-let-7c-5p. Interestingly, miR-195 was elevated in triple-negative BC (75). Using global miRNA profiling of 23 female BC patients from Saudi Arabia, Hamam et al. were able to identify several circulating miRNAs, including hsa-miR- 308 1290, hsa-miR-188-5p, hsa-miR-1225-5p, hsa-miR-4270, hsa-miR-1202, hsa-miR-1207-5p, hsa- miR-4281, hsa-miR-642b-3p, and hsa-miR-3141. Remarkably, they could concentrate and isolate more miRNAs from the patients’ blood samples using a speed vacuum method. The isolated miRNAs were used as a biomarker signature for early-stage detection of BC (116). However, Hamam et al. reported that hsa-miR-155 and hsa-miR-21 were not significantly elevated in the patients’ plasma samples, although reported in other cohort studies. Moreover, Alshatwi et al. found that the miRNAs hsa-miR-146a, hsa-miR-499, and hsa-miR-196a2 were significantly upregulated the blood of 92 patients with BC from Saudi Arabia. Additionally, they identified unique genotypic miR-423 (TT) variances in 100 Saudi BC patients compared with matching healthy individuals (117). These genetic variances were associated with metastases and advanced- stage BC (118). Another recent study by Alajez et al., which aimed to discover miRNA biomarkers in samples from Saudi patients to predict metastases (119), reported the downregulation of seven of the miR-200 family of miRNA, including hsa-miR-200a, b, and c in patients with metastasis compared with the primary tumor samples. Other miRNAs identified included hsa-let-7c-5p, hsa- miR-214-3p, hsa-miR-210-3p, and hsa-miR-205-5p, which were also downregulated. The miRNA, hsa-miR-205-5p, was found to modulate Myc, forkhead box O1 (FOXO1), and the amphiregulin (AREG) pathways. Additionally, the expression of hsa-miR-214- 3p and hsa-miR- 205-5p was correlated with a low survival rate. Furthermore, the global miRNA expression profile confirmed the upregulation of hsa-miR-146a, confirming the findings of Alshatwi et al. reported in Saudi plasma samples, along with other miRNAs such as hsa-miR-150-5p, hsa-miR-155-5p, and hsa-miR-142-5p.

### 2.9 Clinical Application Using Circulating miRNAs in Breast Cancer Patients

To view the latest clinical pilots (August 2021) approved by the Food and Drug Administration (FDA), and used ClinicalTrials.gov and searched for keywords: circulating, miRNAs, and breast cancer. The results have shown eleventh clinical trials with various statuses. However, only five shown are completed; however, these studies did not publish their results. The majority of the studies were completed in France and Italy and one in Poland. Study no. NCT01612871 and NCT03255486 focused on identifying circulating miRNAs correlated with hormonal treatment and neoadjuvant chemotherapy responses in patients’ blood with and without metastases. The other two studies NCT02065908 and NCT02618538 focused on screening women’s blood for early detection of breast cancer. Finally, NCT02065908 to detect cardiotoxicity in BC serum patients because of anthracycline chemotherapy administration.

### 2.10 Strategies in Targeting MicroRNA and Challenges

One of the main rational for targeting miRNA is their ability to crosslink with enormous genes. miRNA’s complex networks can manipulate the cell apoptosis, EMT, chemotherapy resistance, and cell cycle, making it a unique therapeutic target. However, few strategies to interfere with these miRNAs were proposed, such as antisense oligonucleotides, locked nucleic acid, miRNA sponges, recovering tumor suppressor miRNA expression.

#### 2.10.1 Antisense Oligonucleotides

**Figure 2:**
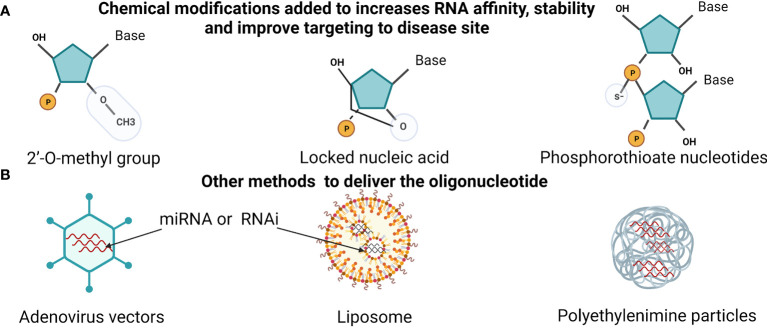
miRNA therapeutics and delivery methods. **(A)** showing the chemically modified oligonucleotide to sustain RNA stability. Such as, adding 2’-*O*-methyl group, linking the 2′-*O* atom and the 4′-C, or adding sulfur to phosphate group (phosphorothioate). **(B)** Methods that are used to increases RNA delivery (miRNA or RNAi). Using adenovirus, liposomes vehicles and synthetic polyethylenimine. Created with BioRender.com.

MicroRNA based treatment is divided into a first and second generation. The first is synthesized as double-strand small RNA that is antisense (RNA mimicry) to target miRNA. In the second generation, a single strand directly targets the mature miRNA strand, antagomirs. Blocking oncomirs using antisense that is modified and specific to the mature miRNA has shown promising results, demonstrated in **Figure 2**. This approach to block miRNAs was enhanced by adding chemical groups to increases RNA affinity to the target by adding the 2’-*O*- methoxyethyl group to the antisense oligonucleotides that also stabilized and protect them from nuclease activity. Hutvágner and his team used this principle to successfully silence an endogenous miRNA let-7 *in vivo* and *vitro (* 120).

Similarly, Esau et al., 2006 conjugated the 2’-O-methyl group, and oligonucleotides phosphorothioate reduced the endogenous miR-122 *in vivo* (121). In 2007, Krutzfeldt and his collage used antagomirs, 2’-O-methyl group, oligonucleotides phosphorothioate, and cholesterol. They injected antagomirs into the tail vein targeting miR-122, which is extremely rich in mice liver. Interestingly, these antagomirs downregulating the endogenous miRNA-122 in 24 hours (122).

#### 2.10.2 Locked Nucleic Acid

Competing with the antagomirs, the Elmen team also targeted the endogenous miRNA-122 but used a ribose ring locked with methyl group by connecting the 2′-*O* atom and the 4′-C atom. That gave the molecular more affinity, stability at a significantly lower dose than the conjugated cholesterol by the Krutzfeldt investigation team, illustrated in **Figure 2** (123).

#### 2.10.3 MicroRNA Sponges

“miRNA sponges” was first presented in 2007 by Margaret and colleges. The term “miRNA sponges” is used to describe a vector with a robust mammalian promoter that transcript competitive tandem binding sites to a specific miRNA or a complimentary seed sequence for a family of miRNA. A seed sequence or region is the 2- 8nt bases at the 5’ of the miRNA complementing a specific subset of targets (mRNA) (124). This seed region is critical and based on it miRNA family is classified. It was successfully introduced in a transgenic animal (*Drosophila* microRNA sponge), demonstrating the miRNA functionality *in vivo* (125).

#### 2.10.4 Recovering Tumor Suppresser MicroRNA Expression

One of the hallmarks of cancer is the inactivation of tumor suppressors. As we showed, many miRNAs can function as tumor suppressors by targeting another oncogene mRNA. Using the same principle as the antisense oligonucleotide, rather than targeting to repress the miRNA replaces the lost one, miRNA mimic. Introducing miR-15a and miR-16 induces cell arrest and apoptosis in prostate tumor xenografts (126). Similarly, miR-29b oligonucleotide on the acute myeloid leukemia xenografts model activates cell death (127). Another method to deliver the oligonucleotide is using viral vectors. Adenovirus vectors do not intergrade their genome host, making it a great model for providing oligonucleotide. Reducing toxicity and with highly transduction efficiency and accuracy (128). In 2009, Kota and colleges successfully overexpressed miR-26a significantly omitted in hepatocellular carcinoma cell lines using an adenovirus- associated vector. MiR-26a target transcript activates cell cycle, cyclin D2 and E2 making it a great target to investigate *in vivo*. Transduction miR-26a in mouse animal models for hepatocellular carcinoma protected the mice from liver cancer (128).

#### 2.10.5 MicroRNA as a Therapeutic Target and Challenges

Using miRNAs as anti-cancer therapy or targeting their genes could serve as novel treatment strategies to overcome several cancer phenotypes, such as drug resistance and metastases. miRNAs could be targeted by using antisense oligonucleotides specific to certain oncomirs to block their oncogenic activity. Additionally, miRNAs that act as tumor suppressors could be developed as novel therapeutic modalities. To view the latest clinical trials, we used ClinicalTrials.gov and the drug name. A handful of approved miRNA by the FDA had reached the clinical trials, described in **Table 2**. However, over 50 RNA interferences (RNAi) drug treatments are ongoing or completed the clinical test with similar methods in delivery and mechanism as miRNA, explained in **Figure 2** (129). For example, the first RNAi mediated drug that reached the market by Alnylam Pharmaceuticals was for hereditary transthyretin-mediated amyloidosis disease in 2018 and RNAi drug for acute hepatic porphyria in 2019 (130).

**Table 2:** Summary of miRNAs drugs FDR-approved in clinical trials.

Drug/company	miRNA	Delivery method	Disease	Phases, status, and Clinical trial no.
MRX34 (Mirna Therapeutics)	miR-34 mimic	Liposomal nanoparticle	Advance solid tumor	Phase I, terminated due to severe immune response, NCT01829971 (134)
MesomiR-1 (EnGeneIC)	miR-16 mimic	TargomiRs	Malignant Pleural Mesothelioma and NSCLC	Phase I, ongoing, NCT02369198 (135)
MRG-201 (miRagen Therapeutics)	miR-29 mimic	Cholesterol- conjugated	Keloid	Phase I, ongoing, NCT02603224
MRG-106 (miRagen Therapeutics)	miR-155antag omir	Locked nucleic acid	Cutaneous T-cell Lymphoma, Mycosis Fungoides, Chronic Lymphocytic Leukemia, diffuse large B-cell lymphoma and T-cell leukemia/lymphoma	Phase I, ongoing, NCT02580552
AZD4076 (Regulus Therapeutics)	miR-103/107 antagomir	GalNAc	Type 2 diabetes and non-alcoholic fatty liver	Phase I/II, ongoing, NCT02612662 and NCT02826525
Miravirsen (Hoffmann-La Roche)	miR-122 antagomir	Locked nucleic acid	Chronic hepatitis C	Phase I/II, varies, NCT01646489, NCT01200420, NCT02508090, and NCT02452814

NCT00000000 (ClinicalTrials.gov identifier).

**Figure 3:**
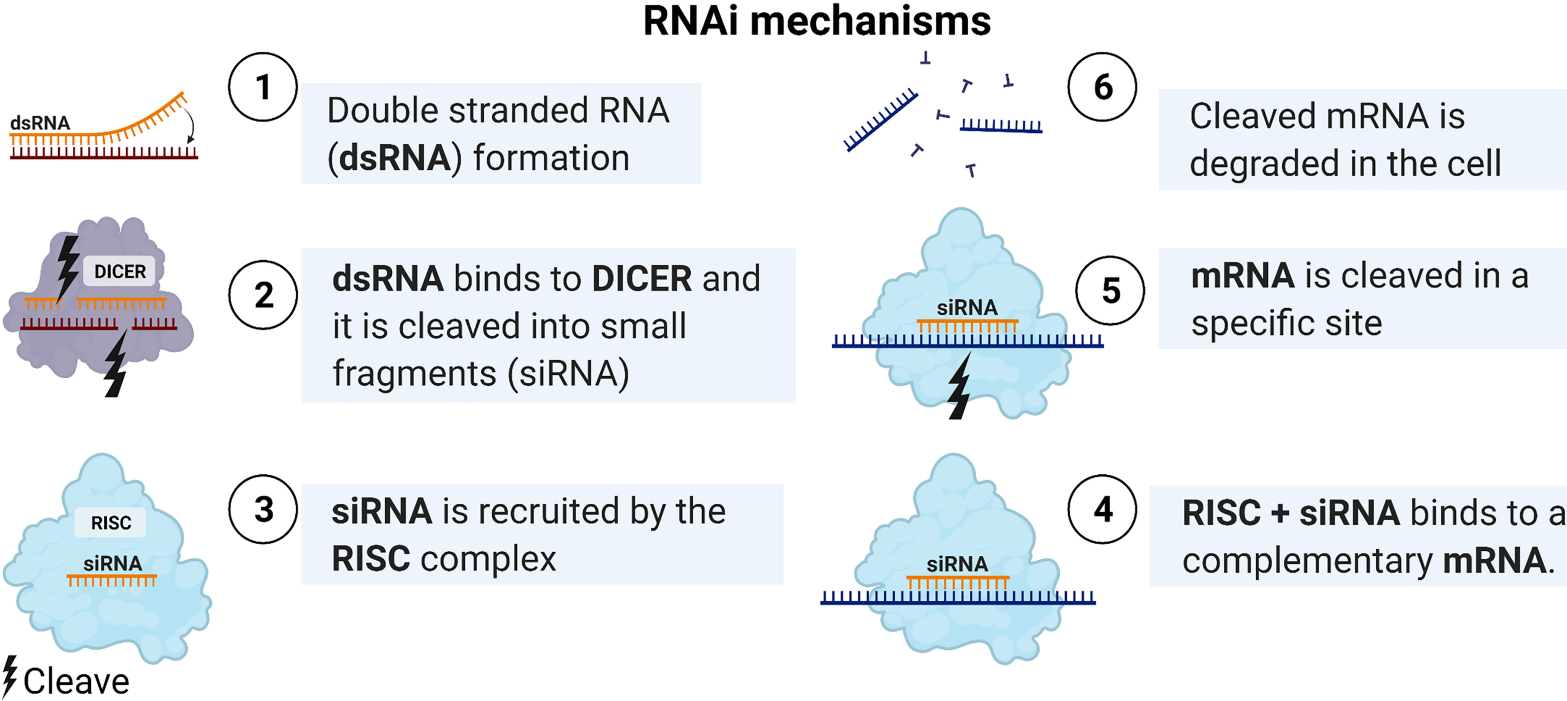
RNAi mechanisms. After transfecting the double strand RNA, it is cleaved by DICER to form a smaller double-stranded miRNA. Then loaded as a guide for the miRNA-induced silencing complex (miRISC) to target the mRNA. Created with BioRender.com.

Nevertheless, newer or enhanced delivery methods have been developed that increase the efficiency of miRNA therapy reached clinical trials, such as neutral lipid emulation, liposomes, and synthetic polyethylenimine demonstrated in **Figures 2** and **3** (131, 132). Moreover, a system using bacterium-derived 400 nm particles conjugated with EGFR antibodies to deliver miR-16 mimics ongoing clinical trials, known as TargomiRs. Similar, small RNA can be linked to N-acetyl-D-galactosamine (GalNAc), another system that uses the cell endocytosis mechanism in phases 1 and 2 and continuing, displayed in **Table 2**. However, the miRNA therapy field is still facing many challenges and young in the therapeutic area similar to RNAi therapeutics, including delivery, stability, off-target effects, and safety (133). Furthermore, miRNAs detected in Saudi Arabia BC patients are still limited, and further studies are needed to provide clinicians with guidelines before applying miRNA-based treatments.

### 2.11 Conclusion

To date, there have been significant scientific research findings demonstrating the functionality of miRNAs as markers for the prediction, prognosis, and diagnosis of cancer. In addition, accumulating evidence suggests that the suppression of oncomirs or stimulation of tumor-suppressive miRNAs could be used to develop novel treatment strategies, such as RNAi and miRNA-based therapeutics (133). These technologies will significantly lower diagnostic costs, robust the clinical treatment methods, and add molecular targeting to enhance patient prognoses. However, this field is still evolving and still facing many challenges that need to be solved. For example, more profiling for miRNA and identifying their targets, reducing the off-target toxicity, creating a better chemical modification increases cellular uptake to the oligonucleotide, viral delivery efficiency, and safety. However, many preclinical tests are shown promising results as researchers are currently focusing on these issues, and pharmaceutical companies show interest in this area presentation opportunities to grow.

## Author Contributions

The author confirms being the sole contributor of this work and has approved it for publication.

## Conflict of Interest

The author declares that the research was conducted in the absence of any commercial or financial relationships that could be construed as a potential conflict of interest.

## Publisher’s Note

All claims expressed in this article are solely those of the authors and do not necessarily represent those of their affiliated organizations, or those of the publisher, the editors and the reviewers. Any product that may be evaluated in this article, or claim that may be made by its manufacturer, is not guaranteed or endorsed by the publisher.
